# A Case of New Familiar Genetic Variant of Autosomal Dominant Polycystic Kidney Disease-2: A Case Study

**DOI:** 10.3389/fped.2015.00082

**Published:** 2015-10-09

**Authors:** Tetiana Litvinchuk, Yunxia Tao, Ruchi Singh, Tetyana L. Vasylyeva

**Affiliations:** ^1^Department of Pediatrics, Texas Tech Health Sciences Center, Amarillo, TX, USA; ^2^Department of Internal Medicine, Texas Tech Health Sciences Center, Amarillo, TX, USA

**Keywords:** ADPKD, *PKD2* gene, *PKD1*gene, polycystin-2, kidney disease, genetic variant

## Abstract

**Background:**

Autosomal dominant polycystic kidney disease (ADPKD) is characterized by renal cyst formation due to mutations in genes coding for polycystin-1 [*PKD1* (85–90% of cases), on ch 16p13.3] and polycystin-2 [*PKD2* (10–15% of cases), on ch 4q13-23] and *PKD3* gene (gene unmapped). It is also associated with *TSC2/PKD1* contiguous gene syndrome. ADPKD is usually inherited, but new mutations without a family history occur in approximately 10% of the cases.

**Case presentation:**

A 17-year-old boy was followed up for bilateral cystic kidney disease, hypertension, and obesity since he was 13 years old. The diagnosis was an accidental finding during abdominal CT at age 13 to rule out appendicitis. A renal ultrasonogram also demonstrated a multiple bilateral cysts. Because of parental history of bilateral renal cysts, *PKD1* and *PKD2*, genetic testing was ordered. Results showed, *PKD2* variant 1:3 bp deletion of TGT; nucleotide position: 1602–1604; codon position: 512–513; mRNA reading frame maintained. The same mutation was later identified in his father.

**Conclusion:**

A smaller number of patients have a defect in the *PKD2* locus on chromosome 4 (resulting in PKD2 disease). There are no known published cases on this familiar genetic variant of ADPKD-2 cystic kidney disease. In this case, the disease is present unusually early in life.

## Background

Autosomal dominant polycystic kidney disease (ADPKD) is an autosomal dominant renal cyst disorder due to mutations in genes coding for polycystin-1 (*PKD1*, on ch 16p13.3) and polycystin-2 (*PKD2*, on ch 4q13-23) and *PKD3* gene (gene unmapped). It is also associated with *TSC2/PKD1* contiguous gene syndrome. ADPKD is usually inherited, but new mutations without a family history occur in approximately 10% of the cases. Here, we describe a case of new genetic mutation, which causes a disease early in life.

## Case Report

A 17-year-old boy was followed up by pediatric nephrologist for bilateral cystic kidney disease, hypertension, and obesity since he was 13 years old. The diagnosis was an accidental finding during abdominal CT at the age of 13 to rule out appendicitis (BUN, 14; Cr, 0.77; electrolytes, WNL).

## Ultrasonogram Finding

**Figure 1:**
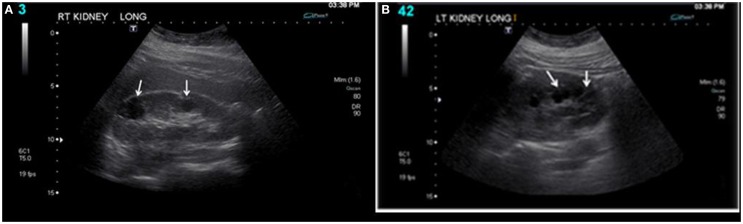
**Bilateral renal cysts: (A) right kidney and (B) left kidney**. At the age of 14, the right kidney measured 11.3 cm × 5.2 cm, and the left kidney measured 11.2 cm × 5.2 cm. There was a cluster of cysts about the upper pole of the right kidney measuring 2.4 cm × 1.9 cm × 3.4 cm and identified cysts about the mid pole region of the right kidney measuring 1.9 cm × 1.6 cm × 1.7 cm and 1.7 cm × 1.2 cm × 2.3 cm. There was a cyst about the mid to lower pole region of the left kidney measuring 1.7 cm × 1.4 cm × 2.0 cm. No hydronephrosis and a good corticomedullary differentiation were observed.

At the age of 14, the right kidney measured 11.3 cm × 5.2 cm (Figure 1), and the left kidney measured 11.2 cm × 5.2 cm. There was a cluster of cysts about the upper pole of the right kidney measuring 2.4 cm × 1.9 cm × 3.4 cm and identified cysts about the mid pole region of the right kidney measuring 1.9 cm × 1.6 cm × 1.7 cm and 1.7 cm × 1.2 cm × 2.3 cm. There was a cyst about the mid to lower pole region of the left kidney measuring 1.7 cm × 1.4 cm × 2.0 cm. No hydronephrosis and a good corticomedullary differentiation were observed. At the age of 17, during follow up, renal ultrasound examination was done. The right kidney measured 11.0 cm × 6.1 cm, and the left kidney measured 11.1 cm × 6.0 cm. There were bilateral cysts, some of which increased in size (2.9 cm × 2.4 cm × 3.1 cm) slightly since the last renal ultrasound examination.

## Impression

Bilateral renal cysts are shown in Figures 1A,B. Because of parental history of bilateral renal cysts, *PKD1* and *PKD2*, genetic testing [familial juvenile nephronophthisis (FJN); Molecular Evaluation] was ordered. This targeted analysis was conducted to determine whether the DNA variants identified in another family member are present in this individual.

## Results

Results showed that *PKD2* variant 1:3 bp deletion of TGT; nucleotide position: 1602–1604; codon position: 512–513; mRNA reading frame maintained. Later, a parental genetic testing was performed and showed the same mutation *PKD2* variant c.1602_1604 3 bp deletion of TGT. There is currently no known published research on this variant.

## Discussion

The occurrence of ADPKD is 1 case per 200–1000 people. Six to ten percent of cases of end-stage renal disease in Europe and USA occur due to this medical condition. ADPKD disease is often present later in life (1). Genetic defects responsible for ADPKD includes, but not limited to, frameshift, deletion, and missense mutations. The most frequent anomaly is on chromosome 16 in the *PKD1* locus that is linked to the alpha-globin gene (85–90% of cases) (2). The other frequent defect is in the *PKD2* locus on chromosome 4 (10% of cases). A few families have a defect unrelated to either locus (3–5). *PKD1* and *PKD2* encode proteins called polycystin-1 and polycystin-2 (6–12).

*PKD1* is a large and composite gene (46 exons) that produces a 14-kb mRNA and encodes protein of over 4000 amino acids in length (7, 8, 11, 13). On the other hand, *PKD-2* is smaller (15 exons) and encodes a protein less than 1000 amino acids in length (11). Mutated proteins are involved in cell differentiation, polarization, proliferation, and membrane transport. Polycystin-1 is localized not only in renal tubular epithelia but also in hepatic bile ductules and pancreatic ducts, leading to cyst formation. It is an integral membrane protein, which is found primarily in plasma membranes but also in the primary cilium and less abundant in adult than fetal epithelia. It is overexpressed in most, but not all, cysts in kidneys from patients with ADPKD and involved in adhesive protein–protein, cell–cell, and cell–matrix interactions (7, 14).

Polycystin-2 is involved in cell calcium signaling (9, 12). *PKD2* gene product is expressed in the distal tubules, collecting duct, and thick ascending limb in normal fetal and adult kidneys. It localizes to the endoplasmic reticulum and not the plasma membrane localized to the primary cilium. Polycystin-1 and polycystin-2 are expressed in similar cellular and subcellular locations, although the overlap is not uniform. Polycystin-1 appears to activate the JAK–STAT pathway, thereby inducing cell cycle arrest (15).

The exact mechanism of cyst formation is not yet understood (16). Abnormalities in renal cilia from mutations in either gene may contribute to renal cyst formation, as PKD1 and PKD2 localize to renal cilia (9). Cysts form in all regions of the nephron, enlarging and expanding throughout life. Normal renal function is maintained until mid-adulthood in most patients.

DNA analysis is the gold standard for the diagnosis of ADPKD-2 because ultrasound is not reliable till a patient is young (17). This is a case of new familiar genetic mutation of PKD2 that probably causes a development of ADPKD in this patient during childhood.

## Conclusion

A smaller number of patients have a defect in the *PKD2* locus on chromosome 4 (resulting in PKD2 disease), and a few families have an undefined defect. In this case, the same familiar genetic mutation was identified. There are no known published cases on this genetic variant of ADPKD-2 cystic kidney disease. In this case, the disease is present unusually early in life.

## Patient Consent

The patient gave consent for this case report to be published.

## Conflict of Interest Statement

The authors declare that the research was conducted in the absence of any commercial or financial relationships that could be construed as a potential conflict of interest.
